# COVID Academic Pandemic: Techno Stress Faced by Teaching Staff for Online Academic Activities

**DOI:** 10.3389/fpsyg.2022.895371

**Published:** 2022-07-28

**Authors:** Mao Zheng, Muhammad Asif, Muhammad Shahid Tufail, Saira Naseer, Shahid Ghafoor Khokhar, Xiding Chen, Rana Tahir Naveed

**Affiliations:** ^1^Faculty of Business Administration, School of Business Administration, Southwestern University of Finance and Economics, Chengdu, China; ^2^Division of Management and Administrative Sciences, University of Education (UE) Business School, University of Education, Lahore, Pakistan; ^3^School of Economics and Management, Nanjing University of Science and Technology, Nanjing, China; ^4^School of Finance and Trade, Wenzhou Business College, Wenzhou, China

**Keywords:** COVID-19, coronavirus, mental health, techno stress, psychological distress, university teachers, online teaching

## Abstract

This paper analyzes the impact of the COVID-19 pandemic on the mental health of the teachers, specifically the techno stress arising in them as a result of issues faced by them in the use of technology when they conduct the online academic activities. It aims to assess the major factors related to the online teaching that specifically adds to techno stress on the teachers during the COVID-19 outbreak. Finally, the study aims to provide suggestions to the policymakers and the management of the universities so that the effect of the COVID-19's on teachers' mental health and the related techno stress can be reduced. This paper is a literature review of the articles on the notion of techno stress on teachers and their mental health by searching the related articles with these terminologies using the renowned search engines of Google Scholar and Web of Science. A combination of the terms such as Coronavirus, COVID-19, mental health, psychological distress, techno stress, and online teaching were used in the article search for the review. The literature has suggested that the COVID-19 outbreak has significantly affected the mental health of the employees in general and specifically, the teachers who are engaged in online academic activities and teaching in the universities. The paper has identified a few factors that are the cause of the techno stress and provides recommendations for the university management and the policy makers for minimizing their negative impact on the teachers, in terms of the techno stress and their mental health. Coronavirus is a new strain of the viruses that has badly engulfed the entire population of the world. It is even now badly rising and causing deaths while this article is in the writing phase. The article has addressed the mental health concerns of the university teachers as they are now working from home using ICT for delivering the lectures and conducting the online teaching and learning activities for the students at their universities. This is a matter of grave importance now and requires immediate attention. Hence, this article broadens the scope of the research on the corona virus and its impact on the university teachers.

## Introduction

The World Health Organization (WHO), on 11 March 2020, announced the corona virus as an outbreak covering the entire globe and declared it to be a pandemic (Salama, 2020). This is a disease that is to date threatening the entire world and its population. It originated from a sea market in the Wuhan province of China in December 2019 (Ruiu and Ruiu, 2020) and since then it has invaded 213 countries of the world and has infected more than 6.3 million population of the world as on 1 June 2020 and has been the cause of nearly 375,000 deaths. The coronavirus is highly infectious that attacks the respiratory system of the human beings and shows symptoms ranging from mild like common cold to severe, like pneumonia and death. This disease is an extension of the Severe Acute Respiratory Syndrome (SARS) and the Middle East Respiratory Syndrome (MERS), however, this is a new strain of virus which had not previously infected the human beings (Király et al., 2020). The main symptoms include dry cough, high fever, body aches, and breathing difficulties. Some individuals may experience varying symptoms as well such as tiredness, pains, runny nose, nasal congestion, diarrhea, and sore throat (Remuzzi and Remuzzi, 2020). A large number of countries have been actively taking precautionary and emergency measures in order to prevent the virus and the infection from spreading. The places where there is crowding of people such as schools, colleges, universities, parks, cinemas, restaurants, and some offices have been closed. Events have been called off to avoid the public gatherings. An enormous number of people have been quarantined, borders have been closed and restriction on travel has caused many canceled flights (Wenham et al., 2020). The medical and healthcare facilities of the countries such as US, UK, China, Italy, France, and many more are suffering from total collapse (Shigemura et al., 2020). This pandemic has shook the world and its economy badly (MacIntyre, 2020). The Organization for Economic Co-operation and Development (OECD) has declared in their latest Interim Economic Outlook that the COVID-19 pandemic has rendered the world with even worst financial crisis after the 2008. The supply chains have been immaterialized badly and many people have been left jobless. In such a time period of layoffs, the employees are finding trouble taking care of the health and the monetary conditions of their families, which is also affecting their mental health badly. A large number of people are suffering from anxiety related behaviors faced with this epidemiological catastrophe (Shigemura et al., 2020). This implies that coronavirus does not only affect the physical health of an individual but is also a risk for their mental health by causing them psychological distress. For instance, take the case of the 37-year-old Japanese government worker who was entrusted to take care of the isolated infected from Wuhan committed suicide (Times, 2020). Even in China and other countries of the world, the number of people with psychological problems, due to COVID-19 are increasing sharply and this has posed another challenge for the health services in these countries (Li et al., 2020).

Since the lockdown implemented strictly by many countries, the closure of the schools and universities in the physical capacity has urged their management to conduct the classes online using the electronic media. This has greatly increased the use of technology and computers. The online technology is a privileged medium by which the institutions can use for achieving their everyday work routines. Even from shopping to working from home to distance learning, this has become the most widely used tool in this crisis (Beaunoyer et al., 2020). However, the factors such as non-availability of internet facility in some areas, lack or limited IT resources, lack of training in the online teaching methodology has added to the stress on the teachers. As a result, the teachers are facing psychological stress and their mental health is being affected (Li and Wang, 2020).

Techno stress is a notion which is relatively underexplored (Brooks and Califf, 2017; Li and Wang, 2020). The research available and existing on this notion have been conducted in the government and the industrial sectors (Marchiori et al., 2019), however, the discussion on techno stress has been limited in the educational sector (Li and Wang, 2020). Some of these studies have focused on the university teachers and the level of techno stress among them, which shows that the teachers are working under stress and pressure when they have to work online in a different and faster more demanding way (Ortagus et al., 2018). Techno stress has many negative consequences that affect the social, physiological, and psychological states of individuals (Atanasoff and Venable, 2017) that can disrupt the sleep patterns and social relations of the individuals, and hence, discourages them to use ICT (Salo et al., 2019) in the already stressful era when the coronavirus pandemic is at its peak. This has a de-motivating impact on them and their work performance is reduced (Hwang and Cha, 2018). Hence, this issue deserves attention from the management of the higher education institutions and the policy makers as the virus is spreading fatly and the lock downs by the governments in their countries are gaining further strictness. Hence, the current paper has the prime objective of assessing the mental health and the techno stress faced by the teachers engaged in the practice of online teaching to their students in this outbreak. This article has novelty since the mental health of the individuals especially that of the teachers specifically from the use of technology has not been investigated before from the perspective of COVID-19. At the end, this article also presents recommendations and suggestions for the management of the educational institutes to address this issue.

## Literature Review

Many studies have investigated the never ending negative impacts of coronavirus pandemic on the individuals and the employees working in any organization, which are also expected to continue long after the pandemic will be gone (Arpaci et al., 2020; Okoro et al., 2020; Yao et al., 2020). Even the healthcare practitioners are getting infected some with the virus and some with the psychological distress caused by the virus (Yao et al., 2020). These stressors include the safety concerns, threat, and risk of being infected from the virus (Xiang et al., 2020). In obesity and the fear of the unknown (Gao et al., 2020), staying isolated and in the quarantine confined to one room or small space (Wang et al., 2020), stigma of being infected and social exclusion (Brooks et al., 2018), and the concerns for job loss and the associated financial issues (Zhu et al., 2020). In addition to these stressors, the mental health of the employees is also being affected badly by the outbreak, which can cause psychological distress in them (Chiu et al., 2020; Lai et al., 2020; Perlis, 2020). The ‘psychological distress' is a specific state of a human being when he/she is suffering emotionally as a result of their difficulty to cope with the life events causing them stress (Cummins et al., 2015). This is shown through a variety of symptoms such as restlessness, sadness, insomnia, and anxiety. The list is never ending, severe psychological distress, if left untreated or non-managed, can lead to major depression, which may eventually make the person commit suicide (Li and Wang, 2020).

The corona outbreak is also a major source of stress for the world population. There are many factors that are causing stress to the employees and hampering their mental health that may even continue after the pandemic as well. The lockdown has urged the need for developing and using the ICT in the universities in a large number of world countries. The potential benefits of online teaching include more autonomy and greater flexibility. It also saves the cost on the transport and may reduce the spread of epidemiological illness (Malik and Javed, 2021). A new mode of online learning has been expanding rapidly owing to the coronavirus, which has caused considerable psychological stress to the teachers who use technology (García-González et al., 2020). The institutions providing higher education have been moving rapidly to innovative teaching using the ICT and online techniques (Markowitz et al., 2018), especially after the arrival and spread of COVID-19. Though a major benefit of ICT is that it has made possible the teaching in such times where the physical classroom was not possible for the fear of the virus spreading among the university students and the staff, it has also contributed to the improvement of the teaching quality. However, on the other hand, the university teachers are facing problems in effectively using ICT as the demand for the skills for using the technology has also increased simultaneously (Li and Wang, 2020). The various factors that are demanding the change in the process of teaching, the changing role of the teacher himself, the work requirements arising due to the use of technology has added to the stress levels of the teachers, who are already forced to put in more time and energy for adapting these changes (Syvänen et al., 2016). As a result, the university teachers are exposed to increased risk and threat of techno stress. Online education, according to Rapanta et al. (2020), requires an established organizational infrastructure that supports online teaching and learning. The emergency remote instruction required under COVID-19, on the other hand, has been frequently devised in a hurry, with little or no infrastructure assistance. Because of the absence of infrastructure, most of the early guidance and assistance for non-expert online professors has centered on the technology tools accessible at each school and deemed suitable to enable the move. However, this “tools-based” approach lacks significant pedagogical clues on how, when, and why to use any of the tools. Thus, many novices online teachers decided to focus on the tools and resources they would use to teach their course topic, whether in person or online that indirectly became the reason for increasing techno stress. Although technology and resources are needed components of online education, teachers' support for students, which includes monitoring their learning processes, is also important. This can be defined as a problem that arises due to the incapability of an individual to cope up with changes in and the new technology and its use (Sellberg and Susi, 2014; Li and Wang, 2020). Previous studies have proved that techno stress has been the cause of many negative side effects on people such as depression, burnout, and decreased levels of productivity and performance (Salo et al., 2019; Brooks et al., 2020).

A study by experts conducting a study on the quality and quantity of information has highlighted that technology affects the cognitions of the online university teachers as the online tasks demand higher intellectual and greater workloads, leading to the mental fatigue (Soria-Oliver et al., 2019) and notable reduction in performance (Saunders et al., 2017). The pace of work and the time pressure associated with the deadlines of the task also because techno stress on the university teachers (Mishra et al., 2020). The time pressure affects the teachers as the time that is available to finish the assigned tasks is less than the time needed to finish it. This causes anxiety in the teachers as the volume of the work spikes and the number of working hours has also increased, which has reduced the personal and the family times as they try to meet the demands of the job (Malik et al., 2017; Pace et al., 2019). Hence, the time dynamics has also played a drastic role in the online teaching, especially in the times of exams or when the workload is double, and time is less to tackle all the tasks well. The mental overload makes the work–life balance more difficult to tackle derived due to the role conflict and they need to extend their working hours from the morning into the hours of the night and the weekend as well (Vargas Rubilar and Oros, 2021). This makes it difficult for the teachers to reconcile the work and family roles. This is a major source of stress for the online teachers as the boundaries between the family and the work remain blurred (García-González et al., 2020).

ICT has changed and revolutionized the way learning and teaching is being done now and has also redesigned the organizational structures of the higher educational institutions and universities (Ortagus et al., 2018). ICT has brought immense benefits to the higher education. It can contribute in improving the learner's ability for enhanced learning experience and better access to the educational resources. But regarding the university teachers, the use of technology brings them more stress as their work load increase, the role gets more ambiguous and the work pattern changes as the knowledge and the required skills need to be upgraded to cover the increasing demands for performance and productivity (Joo et al., 2016). Since the information is continuously being flown into the system, faster response from the teacher is required (Salo et al., 2019). New modes of teaching are being constantly introduced which the teacher must be up-to-date. Hence, this shows that online teaching is more complex and complicated as compared with the traditional form of learning (Hatlevik and Hatlevik, 2018). The operation of using technology is also another challenge for the teachers as few of them know how to operate it accurately and some need training which is not exactly possible due to the coronavirus. Due to these reasons, the university teachers feel anxious and are stressed (Rapanta et al., 2020). Their performance is likely to suffer in such case (Skaalvik and Skaalvik, 2017). Based on these discussions, it can be said that techno stress is not a simple health issue of the employees or teachers, rather it is an issue for the management of the universities and the educational institutions. Studies have shown that by controlling the factors affecting techno stress, the side effects of techno stress can be reduced and minimized, helping the people perform better at their jobs (Joo et al., 2016; Hatlevik and Hatlevik, 2018; Li and Wang, 2020).

## Research Methodology

Grant and Booth (2009) has improvised that the method by which several recent and previous literature studies are examined is considered to be a general literature review. Such review can cover various topics and subjects. For the present article, the subject is the coronavirus COVID-19 which has embarked the entire world and is affecting the whole population. The researchers aim to study specifically the impact of the COVID-19 on the mental health of the teachers who are facing techno stress due to the compulsory conductance of the online classes. The present study has used the narrative form as the main feature of literature review (Grant and Booth, 2009) as used by Hamouche (2020) for similar research. Several searches were made in the world renowned search engines such as Google Scholar and Web of Science with straight terms and combination of terms such as coronavirus, COVID-19, and mental health; COVID-19 and psychological distress, COVID-19, and techno stress of teachers. Research articles, feedback pieces, short remarks, discussions, and review articles published in scholarly publications were among the articles we looked at to gain insight into the impact of COVID-19 on the teachers' mental health. Due to the nature of the investigation, 88 publications were found in a distinct database described earlier. After reviewing tiles, abstracts, and whole pieces, it found 36 irrelevant papers that were repeated from a previous search. Again, research was repeated on the “Web of Science and Google Scholars” database to enhance the search results and double-check the referenced works. Most of the articles that were published in the time frame period of December 2019 and May 2021 were chosen and analyzed since the research on impact of COVID-19 on the employees and teachers are limited, hence, general articles speaking of techno stress were also included in the review. Therefore, in this research, 52 publications are assessed carefully and analyzed for research subjects as well as other factors including the methodologies, settings, and theories employed in the investigations. In addition, this research examines closely connected fields in order to create new research possibilities in the future. By comprehending the research outcomes, the study also examined future direction prospects and research questions. The list of the reviewed articles is included in this article and referenced at the end of the article.

## Discussion and Practical Implications

This article is a general literature review that attempts to evaluate the effect of coronavirus on the mental health and techno stress of the teachers. It presents an insight into the various factors causing stress in the teachers during the unfortunate time of the pandemic. An important aim of this article is to enrich the body of knowledge existing on the influence of COVID-19 on the techno stress of the teachers as a result of the online activities of teaching and academics. The present article also suggests future avenues which the universities and the educational institutions can take so that the teachers can be helped of the techno stress during this outbreak. The theoretical contribution of this study is that there have been very few research articles written on the COVID-19's influence on employees, thus, this tries to widen the reach of this research both from the standpoint of coronavirus and the mental health of workers, particularly teachers. The coronavirus pandemic is highly unpredictable in terms of the contagiousness and the speed of its spreading from people to people and country to country, creating havoc on the economy of the countries and the world as a whole. The institutions have been unable to deal with the concerns and obstacles posed by the pandemic, and one of them is a lack of knowledge, competency, and resources for online teaching activities, which has caused academics and instructors psychological discomfort (Ozamiz-Etxebarria et al., 2021). The management of these institutions and the HR practitioners of the companies must focus on addressing this issue and find accurate and appropriate solutions to protect and safeguard the mental health of their teachers and employees (Leal Filho et al., 2021). This article will prove useful for the firms and add value by helping them understand and recognize the main stressors of the pandemic and improve their employees' mental health by reducing the techno stress. The suggestions put forward by this article will definitely aid the academic leaders and the managers, and they will be able to develop contingency plan to deal with such issues.

### Recommendations

In order to reduce the techno stress faced by the teachers because of the mandatory cancellation of the face-to-face teaching in the universities, it is very important that proper IT infrastructure or resource capacity must be built so that the availability and accessibility of Internet and technology can be ensured. In addition to this, effective training and knowledge updating in terms of the skills and abilities must be made for the teachers. This way the teachers will be able to fully utilize the online learning and teaching tools and gain maximum out of them in this period of crisis using their teaching methodologies without being over stressed. Infect this pandemic has emphasized strongly on the significance of training for the teachers and the up gradation of the online teaching strategies and tools for the educational institutions. The management must establish an online desk help so that technical support could be provided to the teachers and their problems can be solved on the spot. This is the way teachers can be relieved from the stress and they will be able to perform better. The management of the universities must coordinate with the IT staff so that better planning and implementation of the technological interface could be made possible to diminish the techno stress.

## Conclusion, Limitations, and Future Research

The negative impact of the coronavirus on the mental health of the workers, especially, the teachers and the techno stress on them, is the main reason behind conducting literature reviews. Hence, the main aim of the current article is to integrate the necessary information about the virus and its potential effects on the teachers. Hence, this review has the capacity to provide suggestions to the university management and the educators to reduce these effects. However, like any study, this review also suffers from a few limitations. First, despite the quality of the articles provided by the search engines of Google Scholar and Web of Science, the articles selected for the review may suffer from subjective bias. Second, the content of this article is not a systematic literature review, yet it is able to provide well organized and structured information about the virus and its effects that will be useful for the university and the educational institutes. This review can also aid in the development of a research model that can be tested empirically by collecting the data from the real-life subjects. This is a direction for future studies in which an empirical relationship can be investigated between the techno stress, different stressors, and the mental health of employees. The results can be further extended to the era after the pandemic is over so that the lasting effects of coronavirus and the resultant techno stress on teachers can be checked in order to establish a causal relationship. Moreover, the future studies can involve other moderating factors that have not yet been included in this article.

## Author Contributions

MA and MZ: conceptualization, introduction, methodology, interpreted results, and writing—original draft preparation. SN and XC: visualization, validation, conclusion, and writing—original draft preparation. MT and SK: conceptualization, formal analysis, project administration, supervision, finalizes manuscript, review, and editing. RN and SK: literature review, formal analysis, and review and editing. MA, MZ, and XC: writing—original draft preparation, review, and editing. All authors made a substantial, direct, and intellectual contribution to the work and approved it for publication.

## Conflict of Interest

The authors declare that the research was conducted in the absence of any commercial or financial relationships that could be construed as a potential conflict of interest.

## Publisher's Note

All claims expressed in this article are solely those of the authors and do not necessarily represent those of their affiliated organizations, or those of the publisher, the editors and the reviewers. Any product that may be evaluated in this article, or claim that may be made by its manufacturer, is not guaranteed or endorsed by the publisher.
